# Post-COVID-19 Condition: Where Are We Now?

**DOI:** 10.3390/life12040517

**Published:** 2022-03-31

**Authors:** Paula Boaventura, Sofia Macedo, Filipa Ribeiro, Sónia Jaconiano, Paula Soares

**Affiliations:** 1 Institute of Molecular Pathology and Immunology of the University of Porto (IPATIMUP), Rua Júlio Amaral de Carvalho 45, 4200-135 Porto, Portugal; amacedo@ipatimup.pt (S.M.); filipa.ribeiro@i3s.up.pt (F.R.); psoares@ipatimup.pt (P.S.); 2 Instituto de Investigação e Inovação em Saúde (i3S), University of Porto, Rua Alfredo Allen 208, 4200-135 Porto, Portugal; 3 Faculty of Medicine of the University of Porto (FMUP), 4200-319 Porto, Portugal; 4 School of Architecture, Art and Design (EAAD), University of Minho, 4800-058 Guimarães, Portugal; id5928@alunos.uminho.pt

**Keywords:** post-COVID-19 condition, long COVID, SARS-CoV-2, thyroid

## Abstract

COVID-19 is currently considered a systemic infection involving multiple systems and causing chronic complications. Compared to other post-viral fatigue syndromes, these complications are wider and more intense. The most frequent symptoms are profound fatigue, dyspnea, sleep difficulties, anxiety or depression, reduced lung capacity, memory/cognitive impairment, and hyposmia/anosmia. Risk factors for this condition are severity of illness, more than five symptoms in the first week of the disease, female sex, older age, the presence of comorbidities, and a weak anti-SARS-CoV-2 antibody response. Different lines of research have attempted to explain these protracted symptoms; chronic persistent inflammation, autonomic nervous system disruption, hypometabolism, and autoimmunity may play a role. Due to thyroid high ACE expression, the key molecular complex SARS-CoV-2 uses to infect the host cells, thyroid may be a target for the coronavirus infection. Thyroid dysfunction after SARS-CoV-2 infection may be a combination of numerous mechanisms, and its role in long-COVID manifestations is not yet established. The proposed mechanisms are a direct effect of SARS-CoV-2 on target cells, an indirect effect of systemic inflammatory immune response, and a dysfunction of the hypothalamic-pituitary-thyroid (HPT) axis leading to decreased serum TSH. Only a few studies have reported the thyroid gland status in the post-COVID-19 condition. The presence of post-COVID symptoms deserves recognition of COVID-19 as a cause of post-viral fatigue syndrome. It is important to recognize the affected individuals at an early stage so we can offer them the most adequate treatments, helping them thrive through the uncertainty of their condition.

## 1. Introduction

**Figure 1:**
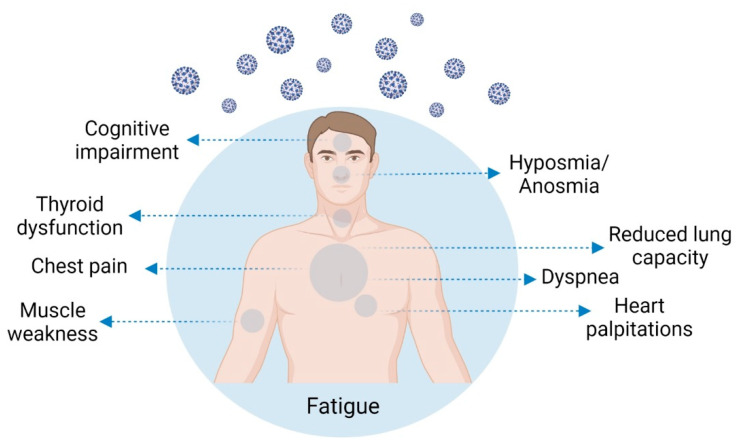
Examples of post-COVID-19 condition chronic complications.

COVID-19 was first described as a respiratory disease, but presently it is considered a systemic infection comprising multiple systems and causing chronic complications [1,2,3] (Figure 1). The pathology results not only from the virus infection but from an aberrant inflammatory host immune response [4]. The immune response has been well described in acute COVID-19 patients, but the lasting consequences of the infection are still not well known [4]. Researchers have been exhaustively surveying the diverse symptoms of long COVID, but until now, no integrated explanation exists for their manifestation [5]. Sykes et al. [6] alerted us to the effects of this poorly known lethal virus, to the societal disruption it has caused, and to the importance it may have in the development of long-lasting physical and mental health symptoms. On the other hand, Sancak and Kilic [1] state that post-COVID-19 condition symptoms can most often be interpreted as somatization; however, the fact that we may not understand them does not mean they are purely psychosomatic [1]. In the study of Xiong et al. [7] in hospitalized patients from Wuhan, a non-infected control group from the general population was used in order to exclude the psychological effects of the long and mandatory isolation period, which caused deconditioning, anxiety, and depression. The authors showed a significant difference between the group of COVID-19 “recovered” patients and the control group, with the latter reporting very few long-term symptoms [7].

## 2. Post-COVID-19 Condition Symptomatology and Prevalence

Protracted COVID-19 infection–related symptoms are common [8,9], but the post-COVID-19 condition [10] (previously referred to as long COVID] is a poorly understood aspect of the current pandemic [9,11]. Compared with other post-viral fatigue syndromes, the symptoms are wider and more intense [12]. An exact definition was recently published by the WHO [10]: typically, symptoms with duration ≥2 months [10,13,14] that cannot be explained by an alternative diagnosis are considered post-COVID-19 condition [10]. Post-acute manifestations may be divided into three categories: (1) residual symptoms continuing after recovery from acute infection; (2) organ dysfunction continuing after initial recovery; and (3) new symptoms or syndromes that appear after initial asymptomatic or mild infection [15].

Over several studies, the frequency of post-COVID-19 condition ranged from 4.7 to 80% (n = 25), occurring between 3 to 24 weeks after the acute phase or hospital discharge [16]. Yong [17], in a study on COVID-19 survivors (n = 10), reported that a post-COVID-19 condition persisted for one to six months in 30−80% of patients. Other studies reported a 35% prevalence of residual symptoms in non-hospitalized patients [18], but around 75–87% in hospitalized patients [6,18,19]. In a cohort of patients followed-up for three to nine months after infection, about 30% maintained persistent symptoms [20]. 

The most frequently reported symptoms, not restricted to severe acute disease [19,21], are profound fatigue [3,6,15,19,22,23,24,25,26,27,28,29,30] or muscle weakness [6,19,29,31], dyspnea [3,6,24,25,28,30], sleep difficulties [6,19,23,28], anxiety or depression [6,19,29], reduced lung capacity [22,32], memory/cognitive impairment (“brain fog”) [23,28,29], hyposmia/anosmia [23], and the inability to fully exercise or work. The most frequent symptom of post-COVID-19 condition is fatigue, which is independent of the acute disease severity or the presence of respiratory problems [33]. A summary of post-COVID-19 condition symptoms, with the frequencies reported and number of patients evaluated, is presented in Table 1. The high variability found between studies is mostly attributable to acute COVID-19 severity, with more frequent symptoms in hospitalized patients compared with patients who suffered from mild or asymptomatic disease.

To better examine this issue, Gaber et al. [27] looked at the effects of COVID-19 infection in healthcare workers, a population with an expected high level of exposure to the virus. They reported a high incidence of infection and a high prevalence of incapacitating post-COVID-19 symptoms, with fatigue commonly reported [27]. Nonetheless, these health workers were unwilling to either seek medical help or take sick leave, despite their struggle to cope with the symptoms [27].

Taquet et al. [40] found a higher incidence of numerous psychiatric disorders in COVID-19 survivors compared with matched patients with influenza or other respiratory tract infections, in a retrospective cohort study using 236,379 electronic health records. The estimated incidence of a neurological or psychiatric diagnosis in the six months following a COVID-19 diagnosis was 33% (95% CI; 33.17–34.07) [40]. Post-COVID-19 condition presents neurological symptoms similar to chronic fatigue syndrome (CFS) and functional neurological disorder (FND] (except for hypogeusia) [41].

Davis et al. [42] conducted an online survey to characterize post-COVID-19 condition in an international cohort (56 countries), tracing the symptoms over 7 months. They found for 91% of the respondents that the time to recovery exceeded 35 weeks; the most frequent symptoms after six months were fatigue, post-exertional malaise, and cognitive dysfunction [42]. According to the authors, their study represents the largest collection of symptoms recognized in post-COVID-19 condition individuals to date (June 2021). More recent studies have shown that persistent symptoms can be found 12 [43] or up to 15 months after recovery from the acute phase of COVID-19 [44]; symptoms are common both in ambulatory and hospitalized patients [44].

## 3. Post-COVID-19 Condition Risk Factors

Post-COVID-19 condition is associated with a weak anti-SARS-CoV-2 antibody response [45], severity of illness [19,34,46,47], female sex [3,5,6,18,19,34,35,45], presence of more than five symptoms in the first week of the disease [3,18,48], older age [18], and presence of comorbidities [18]. Concretely, Fernández-de-las-Peñas et al. [49] reported that the most significant risk factor for developing more post-COVID symptoms was the number of symptoms at hospital admission, which supports the idea that a higher symptom burden in the acute phase of the disease is associated with a higher probability of the post-COVID-19 condition.

Early dyspnea, prior psychiatric disorders, and specific biomarkers (e.g., D-dimer, C- reactive protein, and lymphocyte count) have also been reported as risk factors, even though more research is needed to validate them [3]. Peghin et al. [34] suggested that the constantly elevated titers of the serological response against SARS-CoV-2 may constitute an independent risk factor for the post-COVID-19 condition, since the presence of SARS-CoV-2 IgG antibodies is significantly associated with the condition. Contrarily, Seessle et al. [28] reported that patients presenting at least one post-COVID-19 symptom 12 months after infection did not significantly differ in their SARS-CoV-2 antibody levels when compared with patients without symptoms, although their physical and mental quality of life had significantly decreased.

Interestingly, Townsend et al. [26] showed that significant illness persistence after the COVID-19 acute phase of the disease, affecting health perception, ability to return to work, and the existence of lasting fatigue, appears to be unrelated to the severity of the acute phase, though one would expect to see a difference in post-COVID symptoms between hospitalized and non-hospitalized patients; this hypothesis needs to be verified in upcoming studies [50]. In fact, one puzzling feature of post-COVID-19 condition is that it affects COVID-19 patients at all disease severity levels [3], often affecting patients with a mild acute illness [51]. Studies have shown that post-COVID-19 condition affects even mild to moderate cases [3,52,53] and younger adults (or even children) who did not need respiratory support or hospital or intensive care [3]. Post-COVID-19 condition in children is similar to that seen in adults [54], with symptoms such as a fatigue, dyspnea, myalgia, cognitive impairments, headache, palpitations and chest pain [3,55].

In general, it appears that the ratio for post-COVID-19 condition development is 2:1 in women compared with men, but only until around age 60, when the ratio between women and men becomes similar [14]. 

Post-COVID-19 condition in patients with comorbidities may result from their comorbidity worsening [56].

## 4. Post-COVID-19 Condition Pathophysiology

Different lines of research are trying to explain these protracted symptoms. A persistent immune activation and/or inflammation may contribute to post-COVID-19 condition, which could explain why many patients with mild COVID-19 disease experience chronic persistent symptoms, involving the cardiovascular, nervous, and respiratory systems [57]. In fact, the persistently elevated inflammatory markers observed in long-COVID patients point towards chronic persistence of inflammation [18,58]. 

Shuwa et al. [4] observed lasting alterations in the functional potential of CD8+ T cells from recovering COVID-19 patients up to six months following hospital discharge, which may imply a sustained change in cytokine potential, contributing to a constant inflammatory status [4]. Contrarily, B cell changes seem to be largely restored in convalescence [4]. In a more recent study, Glynne et al. [51] reported that CD8+ EM T cells are diminished for up to 400 days following infection, regardless of symptoms, and CD4+ and CD8+ CMT cell PD-1 levels are augmented following COVID-19 (more marked in post-COVID-19 condition). T-cell dysfunction may promote post-COVID-19 condition pathophysiology similarly to what occurs in autoimmune diseases [3]. It remains to be determined if SARS-CoV-2–specific T cells have the capacity to react against self-antigens [57]. 

Seessle et al. [28] observed several neurocognitive symptoms that were associated with antinuclear antibody titer elevation, pointing to autoimmunity as a cofactor in the etiology of post-COVID-19 neurologic conditions [28]. The autoimmune hypothesis could explain the greater incidence of this condition in women [14,57]. Since thyroid is closely linked to T-cell-mediated autoimmunity, thyroid dysfunction may be important in the pathophysiology of post-COVID-19 condition, as discussed in more detail below [3].

Post-COVID-19 condition has been related to additional characteristics of the innate and adaptive response, involving a weaker initial inflammatory response, with lower baseline levels of C-reactive protein and ferritin [45]. The participation of the immune system in post-COVID-19 condition has been reported in other studies [8,21,57,59,60]. Symptoms such as cognitive dysfunction, persistent fatigue, muscle aches, depression, and other mental health issues are highly associated with an initial immune challenge and/or with a constant dysregulation of the immune system [29,60].

Many neurological anomalies have been described in patients with COVID-19 [41,61], comprising the central and peripheral nervous systems, ranging from mild to fatal, and occurring in patients with severe or asymptomatic SARS-CoV-2 infection [61]. These deferred manifestations may be significant, because they likely affect patients not presenting neurological symptoms in the acute phase [62]. Neurocognitive post-COVID-19 condition symptoms can last for at least one year subsequent to acute infection, diminishing life quality considerably [28].

The involvement of inflammatory cytokines in the etiology of the neuropsychiatric symptoms, reported in current large-scale population-based epidemiological and genetic studies, indicates that these cytokines may have a role in the etiology of the neuropsychiatric symptoms usually observed in patients with post-COVID-19 condition [3,29,60]. This cytokine storm must also be considered as a possible driving factor for the expansion of neuropathies after severe COVID-19 infection, contributing to the chronic pain that appears after acute infection recovery [62]. The augmented cytokine activity, which drives the inflammatory process, disrupts T cell responses, and imposes limitations on neuronal metabolism, may also be an adequate therapeutic target for management and prevention of post-COVID-19 condition [60].

Altered tryptophan absorption and tryptophan-disrupted metabolism have been suggested as key contributors to the enduring symptoms in COVID-19–recovered patients, with numerous studies showing low levels of tryptophan and serotonin in individuals infected with SARS-CoV-2 [63]. Tryptophan is a precursor of melatonin and serotonin, molecules implicated in sleep control and mood disorders, respectively; it is also involved in skeletal muscle mass regulation, a notorious lasting symptom of post-COVID-19 condition [63]. 

Some symptoms may be related to virus- or immune-mediated disruption of the autonomic nervous system, leading to transient or longstanding orthostatic intolerance syndromes [8,31,64,65]. In orthostatic intolerance, the release of epinephrine and norepinephrine causes pronounced tachycardia, which is experienced as palpitations, breathlessness, fatigue, and chest pain, which are common symptoms of post-COVID-19 condition [8]. Alterations in the autonomic nervous system can promote each of these symptoms, theoretically providing a uniting pathobiology for acute, subacute, and lasting sequelae of the infection, and may also be considered as a target for intervention [31]. 

Studies have shown that patients with severe symptoms may have more severe autonomic dysfunction when compared with patients presenting mild symptoms, as indicated by the heart rate variability (HRV) analysis [2], which is a reliable non-invasive tool used to evaluate autonomic modulation [2,64]. Patients with severe symptoms presenting amelioration in autonomic parameters also show enhancements in immune and coagulation functions, as well as in cardiac injury biomarkers [2].

Townsend et al. [66] conducted research to assess if fatigue, the most common symptom following infection, was associated with autonomic dysfunction. No association was found with autonomic dysfunction; the authors found an intense association of fatigue with increased anxiety (*p* < 0.001) in patients without pre-existing diagnoses of anxiety [66].

Another potential cause of post-COVID-19 condition could be the SARS-CoV-2 tropism from the olfactory system into the brainstem, and the consequent persistent, low-grade brainstem dysfunction [17]. SARS-CoV-2 may damage the brainstem through viral invasion, inflammation, and vascular activation [17]. Interestingly, functions of the brainstem and post-COVID-19 condition symptoms have a great degree of overlap [17].

SARS-CoV-2 RNA was found in the brain during autopsy of deceased COVID-19 patients in some studies, but in other studies no SARS-CoV-2 materials were found [17]. This suggests that SARS-CoV-2 neurotropism or brain invasion may happen but not in every patient [17]. The presence of SARS-CoV-2 in the central nervous system has not been directly related to the severity of the neuropathological findings, suggesting that neuronal infection may be only one of the pathways through which SARS-CoV-2 could influence brain function and contribute to some of the long-lasting symptoms of post-COVID-19 condition [29].

Hypometabolism has been reported in post-COVID-19 condition patients; specifically, hyposmia/anosmia was associated with cerebellar hypometabolism [23]. In general, areas of hypometabolism comprised the bilateral rectal/orbital gyrus (including the olfactory gyrus], the right temporal lobe (including the amygdala and the hippocampus extending to the right thalamus], the bilateral pons/medulla brainstem, and the bilateral cerebellum [23]. These metabolic groups allowed distinguishing between patients and healthy subjects with a high power of discrimination. 

Long-term cardiovascular effects of COVID-19 have been described [67]. Vascular events can happen unpredictably in fit patients with mild or asymptomatic COVID-19 infection, even several weeks after the infection [68]. This means that clinicians should remain attentive for post-infective thrombotic sequelae and carefully manage cardiovascular risk factors in convalescent patients, irrespective of the infection severity and the absence of co-morbidities [68]. In post-COVID-19 condition management it is essential to control blood pressure, lipid levels, and obesity after infection with SARS-CoV-2 [53].

Immunological memory of SARS-CoV-2 is not easy to predict [19]. Neutralizing antibody titers at six-month follow-up are significantly lower compared with the acute phase [8,45]. Contrarily, Sette et al. [22] reported data indicating that T and B cell memory and antibodies probably remain for years in most SARS-CoV-2 infected patients. 

As previously mentioned, an additional possibility is that post-COVID-19 condition is caused by an immune system dysfunction that leads the immune system to attack the body, meaning that this condition could be an autoimmune disease [5]. Still, it is precocious to affirm which hypothesis is right and, in fact, it might be the case that each is true in different individuals; preliminary data suggest that post-COVID-19 condition could be various disorders grouped into one [5]. These various disease courses may be traced back to the initial phases of the infection, as shown by the fundamental role of type I IFN responses during the acute phase of SARS-CoV-2 infection [13]. As previously mentioned, the autoimmune hypothesis could explain women’s higher susceptibility to this syndrome [14]. Indeed, women present a stronger immune response for genetic and hormonal factors compared with men; this is a double-edged sword, leading to a more severe outcome of acute infection in men, but to more common autoimmune reactions in women [14].

## 5. Thyroid Involvement in COVID-19

Due to the reported high expression of ACE2, the thyroid may become a target of coronavirus infection, and thyroid involvement in COVID-19 patients has been demonstrated [69]. In fact, SARS-CoV-2 uses ACE2, combined with the transmembrane protease serine 2 (TMPRSS2), as the main molecular complex for the host cell infection [70]. Interestingly, ACE2 and TMPRSS2 expression levels are higher in the thyroid gland than in the lungs [70]. Scappaticcio et al. [70], in their literature review on thyroid dysfunction in COVID-19 patients, presented strong evidence that the thyroid gland and the entire hypothalamic–pituitary–thyroid (HPT) axis may be important targets for SARS-CoV-2 damage. 

Coperchini et al. [71] showed that IFN and, to a minor degree TNF-alpha, regularly increase ACE-2 mRNA levels in normal human thyroid primary cultures. As stated by these authors, the increased pro-inflammatory cytokine levels may enable SARS-CoV-2 penetration in the cells through an additional increase of ACE-2 expression and/or account for the diverse grades of severity of the infection [71]. Nevertheless, additional specific studies are needed to validate this hypothesis [71]. Two main mechanisms account for thyroid function alterations in COVID-19 patients: a direct effect of SARS-CoV-2 on target cells and an indirect effect of the systemic inflammatory immune response [70,72,73,74,75]. A third hypothesis is that dysfunction of the HPT axis causes centrally a decreased level of serum TSH in the infected patients [74]. Changes in thyroid function tests, mostly defined by a TSH level decrease, were described during the acute phase of the infection [69]. These changes have been associated with either destructive thyroiditis or non-thyroidal illness syndrome (NTIS) [69,76]. NTIS, which is defined by low T3 levels, may be caused by any severe systemic disease [76,77]. It occurs due to the diminished conversion of T4 to T3, which is likely elicited by the same factors that cause a decrease in TSH (increase in cytokines and other inflammatory factors) [77,78]. T3 reduction was observed even in mild COVID-19 disease severity, with increased conversion of T4 to reverse T3 [79].

Since manifestations of post-COVID-19 condition include fatigue, and immune dysregulation is one of the proposed mechanisms involved in the condition development, Lui et al. [80] decided to investigate whether thyroid function and autoimmunity play a role in post-COVID-19 condition. They showed, following-up COVID-19 patients, the spontaneous recovery of most thyroid dysfunction observed in the acute phase of the disease, and that incident thyroid dysfunction was a rare situation. Subgroup analysis revealed that symptom recovery occurred more among patients with positive anti-TPO at the time of re-evaluation, suggesting a potential protective role of anti-TPO in post-COVID-19 condition [80]. 

Only a few studies have evaluated the thyroid gland condition in the convalescent stage of COVID-19 [81]. Clarke et al. reported that adrenal and thyroid function was maintained ≥3 months after COVID-19 diagnosis, even though an important proportion of patients suffered from chronic fatigue [82]. 

Campi et al. [69] found a temporary situation of low TSH with normal T4 and low T3 levels in patients hospitalized for SARS-CoV-2 infection, which was inversely associated with C-reactive protein, cortisol, and IL-6, and positively associated with normal Tg levels. These authors stated that this temporary change was probably due to the cytokine storm induced by the virus, with a direct or mediated impact on TSH secretion and deiodinase activity, and probably not to a destructive thyroiditis. The THYRCOV study offers early evidence that patients with acute SARS-CoV-2 infection with thyrotoxicosis have statistically significantly higher levels of IL-6 [83]. In a short-term follow-up, Pizzocaro et al. [84] showed a spontaneous normalization of thyroid function in most infected patients with SARS-CoV-2-related thyrotoxicosis. Nevertheless, these authors stated that long-lasting studies are needed, since they found a frequent thyroid hypoecogenicity pattern in the ultrasonographic evaluation of these patients, which may predispose them to late-onset thyroid dysfunction development [84].

Subacute thyroiditis related to COVID-19 typically presents without pain and with thyrotoxicosis, which in some cases is followed by hypothyroidism [73,85]. Subacute thyroiditis was reported in 13 cases (in 10 papers), detected 7 weeks before to 7 weeks after the diagnosis of COVID-19 [76], so only some of these cases could be compatible with post-COVID-19 condition. 

Dworakowska et al. [86] stated that clinicians should be aware of subacute thyroiditis likelihood, particularly in the early weeks or months after even mild COVID-19 infection. Subacute thyroiditis might be considered as a late complication of SARS-CoV-2 infection, since it frequently arises a few weeks after the upper respiratory tract infection [75,87]. It may be difficult to promptly diagnose this due to a potential lack of classic symptoms and to shared clinical features between COVID-19 and thyrotoxicosis [88].

Recently, Trimboli et al. [89] conducted a systematic review of subacute thyroiditis in COVID-19 patients, concluding that the size and quality of published data are poor, with only case reports and case series being available. According to the authors, and based on these evidence-based data, subacute thyroiditis cannot yet be considered as a direct or common complication of SARS-CoV-2. Still, this assumption might change in the future, considering the fast worldwide diffusion of SARS-CoV-2 and its variants [89].

Even though clear evidence is missing, infection of the thyrocyte, thyrotroph, and corticotroph may lead to a decrease in T3, T4, TSH, ACTH, and cortisol levels [76]. HPT dysregulation has been considered, at least in part, responsible for hypothyroidism in COVID-19 [74,76]. Low FT3 levels are independently associated with increased mortality [72,76,90] and disease severity [74,91,92,93] and may be used as a surrogate prognostic biomarker [72,76,90]. 

Sick euthyroidism is the most common thyroid-related issue in COVID-19 follow-up, especially in patients who were hospitalized or were admitted to intensive care units [73]. Euthyroid sick syndrome is a condition characterized by low serum levels of thyroid hormones in patients with nonthyroidal systemic illness who are clinically euthyroid. These alterations were transitory and recovered during follow-up, although long-term follow-up studies on thyroid function are still needed [73]. Asghar et al. [94], who analysed 54 COVID-19 patients, reported severe COVID-19 patterns in those patients who appeared to have euthyroid sick syndrome. They also reported that the precise clinical importance of a low TSH was uncertain. The authors included a cut-off estimation of TSH decline, predicting disease severity; patients with low TSH levels (<0.996 uIU/mL) showed significantly low survival, whereas patients with sufficient TSH (>0.996 uIU/mL) had a higher cumulative survival proportion [94]. One main limitation of this study was its small sample size. Gong et al. [95] reported that critical illness rates (74.07% vs. 37.40%, *p* = 0.001) and mortality rates (51.85% vs. 22.76%, *p* = 0.002) were significantly higher in the low TSH group compared with a normal TSH group. Zou et al. [96] also reported that euthyroid sick syndrome was significantly associated with the disease severity and inflammatory parameters in COVID-19 patients.

An altered thyroid function is a common situation in COVID-19 patients [72,74,97], especially in critically ill patients [92]. Lui et al. [90] reported that approximately 15% of patients with mild to moderate COVID-19 had thyroid dysfunction. Nevertheless, with the data published so far, it is not possible to assume that thyroid diseases are a risk factor for COVID-19 disease [77,98]. Likewise, a higher occurrence of thyroid disease in patients with COVID-19 has not been observed [77,98]. Although COVID-19 is linked to NTIS, it is not clear if it also raises the risk of developing autoimmune hypothyroidism [98]. It is hypothesized that SARS-CoV-2 might directly influence thyroid morphology and function, leading to an aggravation of a pre-existing autoimmune thyroid disease [98]. Additionally, COVID-19 may worsen autoimmune thyroid disease due to its repercussions on the immune system, which may lead to the development of the cytokine storm [98]. Thyroid autoimmunity, evaluated through the presence of anti-TPO antibodies, was common in COVID-19 patients as compared with pre-pandemic controls [99]. 

Recent data demonstrate that thyroid hormones have an important role in protecting the lungs from damage, including those related to SARS-CoV-2 infection [98]. The lung is one of many organs that responds to the thyroid hormone, and the T3 receptor is present in alveolar type II cells [98]. T3 increases cell size and number, stimulates surfactant release, and elevates the sodium- and potassium-ATPase pump activity, increasing the cell capacity to translocate fluid and therefore absorb alveolar oedema fluid [98].

Our knowledge of the thyroid patterns of COVID-19 is still incomplete, as is the etiologic view of COVID-19 and thyroid insults [76,100]. To find direct evidence concerning the nature and cause of thyroid SARS-CoV-2 injury, and the full immune response in those patients with thyroid dysfunction, we need a histologic and cytological examination of the thyroid gland in a wide number of patients [74,76]. Poma et al. [101] detected SARS-CoV-2 in a small number of thyroid specimens (9/25, 36%). Currently, there is no clear statement on the importance of SARS-CoV-2-induced apoptosis in the thyroid dysfunction [102], but in the SARS-CoV-2 outbreak, it was shown that apoptosis plays an important role in thyroid injury [102]. Summing up, thyroid dysfunction secondary to SARS-CoV-2 infection is probably a combination of various mechanisms [74], and its role in post-COVID-19 condition is not yet established. Tutal et al. [103], who performed a systematic review of COVID-19 and autoimmune thyroiditis, considered it reasonable to routinely assess thyroid function, both in the acute phase of the infection and during the convalescence, through serum TSH, T4, and T3 evaluation. Contrarily, based on the assumption that thyroid function usually normalizes on follow-up, Pat et al. [104] did not recommend a widespread thyroid function screening.

## 6. Post-COVID-19 Condition Health Burden and Patient Management

Post-COVID-19 condition (or long COVID) first gained extensive credit among social support groups, and then in scientific and medical communities [3,5,105,106]. It is probably the first illness to be cooperatively identified by patients discovering one another using Twitter and other social media [105]. The term “post-COVID condition” comprises a wide range of organ impairment, and at the moment we do not have enough information to perform a clear diagnosis, to elect a specific treatment, or to indicate a probable prognosis [107]. Some patients may never recover from the illness [52,56], and all age groups are vulnerable [52]. Patients with post-COVID-19 condition are a heterogeneous group, which makes it difficult to advise treatment [108,109]. It is crucial for each patient to find the correct equilibrium between mild activity to avoid deconditioning and not triggering post-exercise malaise [108]. Strategies tackling our levels of stress and/or the stress response, comprising psychosocial intervention, physical exercise, or possibly dietary interventions could be a good approach to counteract some of the negative effects of chronic inflammation [29]. Rebello et al. [110] advanced that physical exercise may counter the neuropsychiatric and endocrine sequelae of post-COVID-19 condition, through the release of circulating factors that mediate the anti-inflammatory response, support brain homeostasis, and increase insulin sensitivity .

Management of post-acute COVID-19 syndrome requires a comprehensive team, including physicians of various specialties (primary care, pulmonology, cardiology, and infectious disease), physiatrists, behavioural health experts, physical and occupational therapists, and social workers, which will address the clinical and psychological aspects of the disease [111]. 

Although still speculative at the present time, there is a considerable body of literature supporting the anti-stress and anti-inflammatory role of certain seated meditations, yoga asanas, and pranayama practices [112]. The possible benefits of these practices encompass wider neuroimmune systems, which is an advantage since we are facing a systemically dysregulating disease in COVID-19 [112]. 

Lastly, we may refer to the role of COVID-19 vaccines in post-COVID-19 condition. Although vaccines prevent death and severe illness, it is not yet clear if they may also prevent post-COVID-19 condition [5]. Small studies have shown that AstraZeneca and Pfizer-BioNTech vaccines were associated with overall improvements in post-COVID-19 condition symptoms [113]. Recently, Antonelli et al. [114] found that the odds of having symptoms for 28 days or more after post-vaccination infection were approximately halved by having two doses of the vaccine.

## 7. Conclusions

It is urgent to better understand this emerging, complex, and puzzling medical condition [16,115]. Post-COVID-19 condition can become a crisis for health systems, which are already facing the challenge of the pandemic [116]. It is essential to be able to better deal with the symptoms of this condition in terms of clinical care, public health, and health resource planning [116]. At the population level, it is necessary to evaluate the burden of post-COVID-19 condition in order to evaluate its impact on the healthcare system and distribute resources in an adequate way [18,48,107,117]. The primary care services, which represent the first approach for patient diagnosis, still have little information or resources to deal with these patients [118]. Patients with post-COVID-19 condition may have a variety of positive and negative healthcare experiences, which can be useful for the creation or adaptation of the healthcare services [119].

The existence of post-COVID symptoms is leading to the recognition of COVID-19 as a cause of post-viral fatigue syndrome, even when the disease acute phase was mild [20,35]. This can help clinicians to organize patient care, namely follow-up visits, rehabilitation, cognitive behavioral therapy, and even simple actions like inclusion of counseling sessions at discharge to diminish patient anxiety about prolonged symptoms [35]. The patients need to be monitored with a systematic protocol, including symptoms of mental and physical health, and specific healthcare programs to support a healthier lifestyle after SARS-CoV-2 infection need to be implemented [120].

There is an urgent need to identify affected individuals early so the most appropriate and efficient treatments may be provided [111,115], helping them to thrive through the uncertainty of their condition [15,121].

## Figures and Tables

**Table 1:** Post-COVID-19 condition symptoms most frequently reported.

Post-COVID-19 Symptoms	Number of Patients Included in the Study	% Patients Suffering from Symptom/References
Fatigue	596, 177, 538, 270, 138, 3065, 134, 242, 115, 143, 96, 1733, 5440, 384, 287	13.1% [34], 13.6% [20], 28.3% [7], 34.8% [25], 39.0% [27,30], 39.6% [6], 41.7% [35], 47% [26], 53.1% [36], 56.3% [28], 63% [19], up to 65% [16], 69% [37], 72.8% [38]
Persistent breathlessness /dyspnea	596, 3065, 287, 270, 96, 138, 143, 384, 134, 5440, 35	6.0% [34], 23.2% [30], 28.2% [38], 34.0% [25], 37.5% [28], 40.0% [27], 43.4% [36], 53.0% [37], 60% [6], up to 61% [16], 80% [23]
Myalgia /muscle weakness	277, 242, 134, 1733	19.6% [25], 35,1% [35], 51.5% [6], 63% [19]
Anxiety	287, 402, 134	38.0% [38], 42% [39], 47.8% [6]
Sleep disturbance	1733, 96, 134, 35, 138	21.1% [35], 26.0% [19], 26.0% [28], 35.1% [6], 40.0% [39], 46% [23], 49% [27]
Joint pain	277, 143, 287	19.6% [25], 27.3% [36], 31.4% [38]
Headache	242, 270, 3065, 287	19.0% [35], 19.8% [25], 23.4% [30], 28.6% [38]
Chest pain	596, 242, 538, 143, 287, 35, 5440	0.8% [34], 10.7% [35], 12.3% [7], 21.7% [36], 28.9% [38], 34.8% [23], up to 89% [16]

## Data Availability

Not applicable.
